# Continuous physiological monitoring using wearable technology to inform individual management of infectious diseases, public health and outbreak responses

**DOI:** 10.1016/j.ijid.2020.05.086

**Published:** 2020-07

**Authors:** Damien K. Ming, Sorawat Sangkaew, Ho Q. Chanh, Phung T.H. Nhat, Sophie Yacoub, Pantelis Georgiou, Alison H. Holmes

**Affiliations:** a NIHR-Health Protection Research Unit in Healthcare Associated Infections and Antimicrobial Resistance, Imperial College London, UK; b Department of Family Medicine, Hat Yai Regional Hospital, Thailand; c Oxford University Clinical Research Unit (OUCRU), Ho Chi Minh City, Viet Nam; d Centre for Tropical Medicine and Global Health, University of Oxford, UK; e Department of Electrical Engineering, Imperial College London, UK; f Centre for Antimicrobial Optimisation (CAMO), Imperial College London, UK

## Abstract

Optimal management of infectious diseases is guided by up-to-date information at the individual and public health levels. For infections of global importance, including emerging pandemics such as COVID-19 or prevalent endemic diseases such as dengue, identifying patients at risk of severe disease and clinical deterioration can be challenging, considering that the majority present with a mild illness. In our article, we describe the use of wearable technology for continuous physiological monitoring in healthcare settings. Deployment of wearables in hospital settings for the management of infectious diseases, or in the community to support syndromic surveillance during outbreaks, could provide significant, cost-effective advantages and improve healthcare delivery. We highlight a range of promising technologies employed by wearable devices and discuss the technical and ethical issues relating to implementation in the clinic, focusing on low- and middle- income countries. Finally, we propose a set of essential criteria for the rollout of wearable technology for clinical use.

## Highlights

• Wearable devices will play an increasing role in outbreak management.
• An increasing range of actionable physiology can be captured non-invasively.
• Community deployment for syndromic surveillance could assist public health measures.
• Privacy and data ownership issues in the use of wearables still need addressing.

## Introduction

Infectious diseases with significant global impact, including sepsis, malaria, dengue, and emerging viruses, commonly give rise to fever and differing degrees of systemic involvement resulting from an underlying inflammatory process. Although presentations differ, severe illness can manifest in common physiological derangements such as hemodynamic shock or hypoxia – their management will often require organ support interventions delivered in intensive care settings. Efficient use of these resources requires a system with the capacity to recognize early physiological derangements in deteriorating patients. However, in many acute healthcare settings, particularly in low and middle-income countries (LMICs), implementing an effective, rapid, patient warning system can be challenging (Beane et al., 2018). Limitations on infrastructure, clinical staffing, high disease prevalence, and workload factors can limit healthcare effectiveness (Schultz et al., 2017).

Clinical studies have consistently demonstrated the need for timely medical intervention; delayed recognition of illness results in adverse outcomes and increased costs (Judd et al., 2014, Singer et al., 2016). There is additionally a complementary need to strengthen frontline community health services to allow for identification and appropriate referral of unwell patients. Maintaining patient referral pathways protects the delivery of acute healthcare through improving healthcare integration (Topp et al., 2018). The importance of health system resilience and preparedness was exemplified in the 2014 West African Ebola epidemic (Siedner et al., 2015) and will be vital in bolstering responses for the 2019 COVID-19 pandemic (Kandel et al., 2020).

Continuous patient physiological monitoring involves measuring parameters such as pulse, electrocardiography, blood pressure, oxygen saturation, and respiratory rate through a combination of invasive and non-invasive methods. These parameters guide clinical decision-making in many developed healthcare settings; however, their applicability to LMICs is less well defined. Expensive equipment requires operator training, and low clinical staffing numbers might mean that a direct translation is unworkable (Turner et al., 2019). At the same time, advances in electrical hardware design and data processing methods have enabled technology capable of detecting multiple aspects of physiology in a continuous, minimally-invasive fashion. Devices that can be worn on the body surface for prolonged periods hold promise, particularly for infectious disease clinical management in LMIC settings because of their relatively low costs and potential for connectivity. In conjunction with rapid diagnostics, their use could strengthen community healthcare and deliver decentralized care, informing public health responses in outbreak settings.

In our article, we illustrate the leading "wearable" device technologies, describe potential roles in healthcare, particularly in LMICs, and discuss challenges associated with widespread implementation.

## Wearable technologies

We define a wearable healthcare device as a technology that can be appropriately placed on the body by the end-user and can monitor relevant aspects of health at an actionable standard. These devices may obtain data through tracking physiological parameters non-invasively, or sense substrates from body sites in a minimally-invasive fashion. In recent years, the number of consumer-grade wearables such as the FitBit™ or Apple Watch™, and devices designed specifically for healthcare use have increased significantly (Dunn et al., 2018). The primary sensing modalities are summarised in Table 1, and as follows:

**Table 1:** Main sensing technologies employed in healthcare wearable devices, and selected examples of devices which are available or in development and have to undergo in-human validation. Note several wearables that incorporate multiple sensing modalities.

Sensing modality	Physiological sensing parameters	Device examples and placement location	Selected clinical studies	Country of clinical testing	Comments and limitations
Photoplethysmography (PPG) – reflective and transmissive	Pulse waveform - SpO2 and heart rate reliably derived. Additional parameters: Blood pressure, respiratory rate, heart rate variability, hematocrit are under development.	Everion (arm)	None but monitoring in healthy volunteers showed acceptability (Beeler et al., 2018), and validation with Holter monitoring (Barrios et al., 2019).	Switzerland	Prone to artifact created by motion or skin perfusion state. Accuracy varies according to site of measurement with best results from finger.
		Empatica E4 (wrist)	A study of 69 patients with epilepsy using motion and electrodermal activity sensors on the device showed the ability to characterize generalized seizure activity (Onorati et al., 2017).	USA	
		ViSi Mobile (finger)	Pilot study on 20 hospitalized patients showed reasonable concordance with early warning scores derived from nurse-obtained measurements (Weenk et al., 2017).	Netherlands	
Electrical impedance	Respiratory rate.	Sensium* (chest wall)	Pilot study on 61 patients showed general agreement in heart rate and respiratory rate compared with conventional bedside monitoring (Hernandez-Silveira et al., 2015), but a survey of 50 medical patients showed a lack of agreement with nurse-obtained measurements (Granholm et al., 2016).	United Kingdom and Denmark	Subject to motion artifact. Volume status assessment through bioimpedance currently a research tool.
		Equivital^†^ (chest wall)	None but healthy volunteer validation study with Holter monitoring (Akintola et al., 2016).	Netherlands	
Electrocardiography	ECG activity – heart rate and rhythm Respiratory rate is possible.	Lifetouch^▲^ (chest wall)	Abstract presentation on use in 19 hospitalized patients at risk of liver decompensation showed relationships with clinical severity and inflammation (Chatterjee et al., 2014).	United Kingdom	Arrhythmias such as atrial fibrillation may affect the accuracy of respiratory rate measurement, although signal processing methods possible.
		VitalPatch^▲^ (skin)	A randomized clinical trial of 20 patients in usual care or home monitoring showed wearable use associated with lower costs (Levine et al., 2018).	USA	
		Zephyr*, ^▲^ (chest wall)	Initial validation studies in 22 healthy participants over 45 minutes show good relationships for heart rate, respiration rate, and motion but moderate relationships with skin temperature (Johnstone et al., 2012)	USA	
		BioStamp (chest and leg)	Validation in 30 healthy volunteers over two days comparing heart rate, heart rate variability, respiratory rate and motion with other measurement modalities (Sen-Gupta et al. 2019)	USA	
Biosensor	Various: glucose, lactate, antibiotic concentrations	Glucose e.g. Dexcom G6 CGM	The use of continuous glucose monitoring improved glycemic control in type I diabetes with high acceptability (Beck et al., 2017).	USA	Commercially available devices not available yet requires minimally-invasive placement, e.g., on skin
		Microneedle platform (skin patch – ongoing research)	Healthy volunteer levels of penicillin V in interstitial fluid detected by wearable showed similar pharmacokinetics to free drug measurement (Rawson et al., 2019).	United Kingdom	
Additional modalities: * ECG and temperature probe; ^†^ ECG, motion, temperature and PPG probe; Motion detection					

## Photoplethysmography

Photoplethysmography (PPG) utilizes light reflected from the skin surface to non-invasively characterize features of the underlying circulation. PPG is employed widely as pulse co-oximetry for assessing oxygenated/deoxygenated hemoglobin proportions to derive oxygen saturation (SpO_2_) in healthcare settings. Its use in an ambulatory and wearable context has been explored and may aid in the management of chronic respiratory conditions (Buekers et al., 2019). Also, there is some evidence that parameters, including pulse pressure, respiratory rate, and pulse variability as a proxy of autonomic status, can also be calculated through analysis of the pulse waveform morphology (Allen, 2007).

Utilization of low-cost pulse oximeters coupled with machine learning-driven analysis has been shown to predict the onset of autonomic dysfunction in hand-foot-mouth disease (Abebe Tadesse et al., 2020) and tetanus. There is also promising research in estimating fluid status using the PPG waveform alone (Convertino et al., 2013), and this has been applied to pediatric patients with dengue shock syndrome (Moulton et al., 2016). The extension of PPG analysis for the non-invasive measurement of hematocrit is possible (Phillips et al., 2012) and represents an ongoing research area in dengue (Rodriguez-Manzano et al., 2018). Although PPG is acceptable by patients, measurement accuracy can be subject to motion artifacts and local perfusion changes. Novel data processing methods that compensate for weak signals can be used to improve signal reliability (Jarchi et al., 2019).

## Detection of electrical activity and impedance

Respiratory rate is a sensitive parameter for patient deterioration (Goldhill et al., 1999), and measurement of chest expansion can be done by transthoracic impedance. Lung expansion results in a voltage change across the chest surface, and the placement of powered-electrodes on the skin can capture changes in electrical impedance. Similarly, electrocardiography (ECG) readings can be measured and analyzed to derive respiratory rate given the variation of heart rate with respiration (Charlton et al., 2016). In the form of a small adhesive skin patch on the chest, wearables have undergone clinical studies but with variable performance (Downey et al., 2019, Breteler MJM et al., 2020). The assessment of fluid volume status (Covic et al., 2017) and cardiac output (Cotter et al., 2004) through bioimpedance is clinically feasible and remains an ongoing research area.

## Biosensing

Biosensing refers to the specific detection of substrates in the body through a variety of methods, including enzymatic, antibody, and electrochemical approaches (Kim et al., 2019). Developments in microfluidics also mean that laboratory analyses traditionally performed on blood samples can utilize small volumes of accessible-fluids such as saliva or sweat at the point-of-care. A particularly promising avenue of biosensing has been pioneered in continuous glucose measurement of interstitial fluid (ISF). The ISF is in direct communication with blood and allows for unrestricted diffusion of small molecules. The use of the continuous glucose monitor over 24 weeks resulted in improvements in glycemic control in patients (Beck et al., 2017). A phase I study using a microneedle platform – a small minimally-invasive skin patch to measure ISF beta-lactam concentrations – also demonstrated promising results with good acceptability in healthy volunteers (Rawson et al., 2019). Continuous detection of substrates like lactate using the same microneedle platform is possible (Bollella et al., 2019) and could be utilized to guide therapy in malaria (Aramburo et al., 2018), sepsis (Gu et al., 2015) and dengue (Yacoub et al., 2017) without laboratory testing.

Other existing sensor modalities that utilize 3-axis accelerometry, skin temperature, pressure, and light may also be combined in a multi-modal fashion to improve specificity.

## Wearables in healthcare and infectious disease surveillance

**Figure 1:**
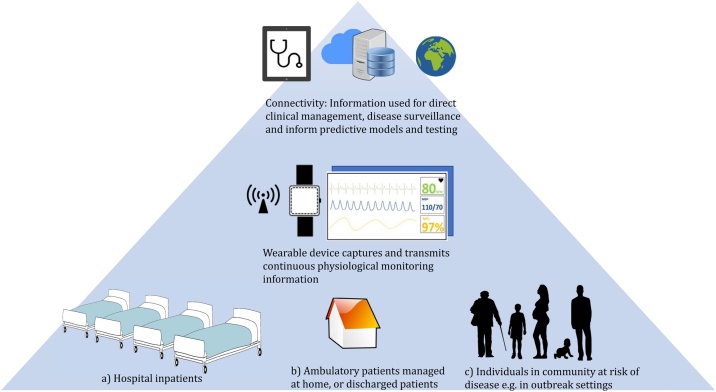
Potential roles for healthcare wearable devices in providing continuous real-time physiological monitoring for: (a) hospital setting to provide early warning in clinical deterioration; (b) ambulatory patient management or follow up of patients discharged at home; (c) deployment to healthy individuals at risk of disease outbreak to provide real-time syndromic surveillance information. The data is connected securely to cloud-based systems and integrated with other sources such as rapid diagnostics and healthcare utilization data. The information is then used for direct patient monitoring, or aggregated at a public health level for surveillance to inform public health measures.

Continuous patient monitoring methods are likely to improve healthcare, providing they are cost-effective and perform at a clinically-appropriate standard (DeVita et al., 2010). We propose that low-cost healthcare wearable devices and rapid diagnostics could fulfill multiple roles for infectious disease management (see Figure 1). Wearables allow for earlier detection of clinical deterioration in hospital: a PPG-based device measuring pulse waveform alone resulted in earlier recognition of shock in trauma patients (Stewart et al., 2016). The use of machine learning algorithms to process continuous physiological signals in real-time could provide predictive alerts directly to clinicians (Daniels and Georgiou, 2019). Biosensor devices which detect specific substrates directly from the body provide additional information for risk stratification, without delays or costs associated with laboratory testing. For infectious diseases with a high potential for nosocomial transmissions such as COVID-19, pandemic influenza, or Ebola, the ability to monitor for early markers of deterioration without direct clinical staff contact and exposure is distinctly advantageous (Steinhubl et al., 2016). Data connectivity from multiple patients to a single point could improve resource and time utilization by identifying sick patients earlier, particularly in a resource-limited setting with low staffing. Efforts using open-source technologies to develop such interfaces are underway (Badgeley et al., 2016). Data from wearable sources can be integrated with other healthcare data through clinical decision support systems to guide specific, appropriate responses, such as antimicrobial therapy (Hernandez et al., 2017).

Healthcare wearable devices acceptable for prolonged use could lead to opportunities in ambulatory patient management and telemedicine. For example, clinical prediction of severe disease in dengue is difficult, and patients who are otherwise well usually return to the clinic for daily assessments, imposing demands on often-strained healthcare systems (Yacoub and Wills, 2014). A PPG-based wearable that can continuously monitor parameters such as pulse, body movement, and hematocrit could be provided for home use, and yield actionable information and identify those at high risk. Non-invasive methods of measurement through wearables are particularly suited for pediatric patients where acceptability is likely higher compared to blood testing.

Connectivity of wearable devices and scope for scalability could also augment the effectiveness of syndromic surveillance. Communication infrastructure exists in most areas of the world, and, together with rapid diagnostics connectivity (Ming et al., 2019), could facilitate studies into the transmission dynamics of infections using location information (Barrat et al., 2014). Implementation of device-based syndromic monitoring, which detects physiological derangements, could be employed in high-risk scenarios. Individuals in the proximity of case clusters for acute respiratory illnesses such as COVID-19, and those at particular risk, could be monitored in the community using wearables. These, in turn, can inform the deployment of appropriate rapid diagnostics or public health interventions. In one example, through the use of heart rate data from a consumer wearable, researchers in the United States were able to forecast influenza-like illness activity, which matched with surveillance data (Radin et al., 2020). Coupling wearables with mHealth technologies such as mobile phone messaging for case reporting, geolocation, and short-range connectivity through Bluetooth might augment contact tracing and map transmission clusters in outbreaks - one such implementation using mobile phones is planned for deployment in the UK for COVID-19 with results to come (NHSX, 2020). The performance of these predictive models is likely to continue to improve with better data acquisition from purpose-built low-cost wearables.

## Challenges to implementation

Significant changes to patient care will likely occur with continuous physiological monitoring through healthcare wearables, however, studies that aim to demonstrate their optimal use are currently lacking. A meta-analysis examining the use of continuous versus intermittent monitoring in developed healthcare settings was unable to demonstrate differences in patient outcomes (Cardona-Morrell et al., 2016). The authors commented on the heterogeneity in outcome measurements and suboptimal designs in some studies: within healthcare systems with multiple safety pathways and redundancies, the added benefit of continuous monitoring may lie in its ability to affect decision-making and increase efficacy in patient management. Defining these outcomes measures and developing accurate evaluation methods will be increasingly important, particularly for LMIC settings where cost-effectiveness is a significant factor. For hospital studies, randomized studies with outcomes that measure the actual clinical benefit of the wearable for the patient, as opposed to examining device performance alone, will be required (Rodger et al., 2012). For the implementation of wearables for ambulatory and surveillance purposes – a systematic multifaceted approach to evaluate these complex interventions will be needed (Moore et al., 2015). Selected examples of healthcare wearable devices utilizing various sensing modalities are presented in Table 1 – these devices have undergone on-human validation but are by no means exhaustive. Of importance to bear in mind is that the clinical design and implementation of these wearables have been conducted in resource-rich settings, which can, in turn, create intrinsic biases in the dataset. We emphasize the need for dedicated studies to be performed in LMICs where applicability and performance can be evaluated appropriately.

The widespread use of healthcare wearable devices will result in the generation of high volumes of individualized, detailed data. Longitudinal datasets will be required to understand and interpret these data. For example, the Precision Medicine Initiative aims to include wearable sensor data linked with health information in over 1 million US participants (Precision Medicine Initiative Working Group, 2015). Issues of privacy and data ownership are paramount – these must be anticipated and addressed directly together with patient partnership. Strict legislation will be required for the use of geolocation and tracking data in syndromic surveillance, given that routine metadata could potentially be linked to individual identification (Perez et al., 2018).

Ensuring the data rights and privacy of the end-user is central to implementation. Appropriate dialogue between developers and end-users to ensure co-design, building on frameworks such as the Fair Information Practice Principles, will be needed. Dedicated studies on wearable use and acceptability should take place across LMICs and diverse cultural settings to identify and explicitly address concerns of relevant users. Supporting user understanding of the purpose of the wearables, facilitating access to their own data and control on the situations *when* monitoring takes place, and *what* types of data is shared should be a prerequisite in design. In particular, balancing individual rights to privacy with public health will be particularly important during specific situations, such as the use of wearables deployed during disease outbreaks.

Broad principles in the use of novel digital health data are beginning to be crystallized by international bodies such as the WHO 2018 Resolution on Digital Health and EU General Data Protection regulation, outlining issues of governance, data minimization, consent, accountability, privacy, and fairness. However, there still remains a need for individual countries to better define their own requirements and establish firm guidelines (Mehta et al., 2020). This has resulted in significant inconsistencies in the ethical frameworks governing the acceptable use of these digital data (Floridi, 2019). Considerable investment in developing expert regulatory capacity is required, given differences in digital health with clinical research and potential societal impact. A requirement for transparency in how the data is used could facilitate this, such as through the development of national digital repositories and algorithms to enable regulatory review after research has been conducted (Samuel and Derrick, 2020).

Regarding hardware development, the use of healthcare wearables remains in relatively early stages of development, with improvements in utility and performance still forthcoming. Power consumption of wearables, limited battery life, and the need for frequent charging may pose issues for effective implementation. Although stand-alone wearables with direct connectivity to WiFi or cellular networks are ideal, current power requirements remain prohibitive. Current wearables that employ local network connectivity with mobile phones using protocols such as Bluetooth Low Energy likely represent a pragmatic compromise in terms of power. Developments in power optimization, such as through algorithms that identify optimal periods of sampling (Culman et al., 2020), may result in more efficient wearables and might be suitable for lower-risk patient groups in the community. In some instances, although individual parameters might be insufficient to differentiate between “normal” and “abnormal” physiology, the integration of different signals through a multi-modal approach may also increase the specificity of early warning systems. Nonetheless, the risk of false-positives generated by warning devices contributes, in turn, to alarm fatigue and can hinder the usefulness of such devices. This is particularly important for commercially-available smart devices where inferior performing products could erode public trust in wearables and affect future acceptability (Bonafide et al., 2017). To allow for monitoring of inflammatory levels or therapeutic drug concentrations in chronic conditions such as tuberculosis or deep-seated bacterial infections, healthcare wearables will need to be used for prolonged (weeks to months) periods. Strategies to incorporate additional non-health, lifestyle or social media functionalities to these healthcare wearables may improve user retention rates. This blurring of boundaries between health and commercial aspects of wearables, particularly in terms of data ownership, again requires a better definition.

With these considerations in mind, we propose a basic set of parameters for healthcare wearable devices to fulfill prior to implementation in Table 2.

**Table 2:** A proposed set of criteria for healthcare wearables

Healthcare wearable devices should be:	
Acceptable for user	Suitable for prolonged use in terms of comfort and functionality; offer the desired level of privacy and data ownership for the user
Actionable data	Provides representative quality data at a level appropriate to inform interventions
Accessibility	Allow for secure data linkage and connectivity through approved bodies
Adaptable	Scalable for rollout. Cost and functionality adaptable to different healthcare resource settings.

## Conclusion

Healthcare wearable devices offer significant advantages for the management of infectious diseases and precision medicine. The technology is undergoing rapid development; however, we show that continuous capture of quality, actionable data from individuals is possible through existing modalities. There are specific challenges for infectious diseases, including the use of geolocation data for surveillance and implementation in resource-limited settings -- ensuring data privacy through legislation will be essential for public acceptability and trust. However, the rollout of healthcare wearables in the near future is likely to improve patient management, providing novel strategies for effectively managing endemic or emerging infections and outbreaks.

## Ethical approval

Ethical approval was not required for this research.

## Declaration of Competing Interest

We declare no conflict of interest.
